# Sequence analysis of pooled bacterial samples enables identification of strain variation in group A streptococcus

**DOI:** 10.1038/srep45771

**Published:** 2017-03-31

**Authors:** Rigbe G. Weldatsadik, Jingwen Wang, Kai Puhakainen, Hong Jiao, Jari Jalava, Kati Räisänen, Neeta Datta, Tiina Skoog, Jaana Vuopio, T. Sakari Jokiranta, Juha Kere

**Affiliations:** 1 Research Programs Unit, Immunobiology, University of Helsinki, and Helsinki University Central Hospital, Helsinki, Finland; 2 Department of Biosciences and Nutrition, Karolinska Institutet, Huddinge, Sweden; 3 Bacterial Infections Unit, National Institute for Health and Welfare, Turku, Finland; 4 Department of Medical Microbiology and Immunology, University of Turku, Turku, Finland; 5 Molecular Neurology Research Program, University of Helsinki, and Folkhälsan Institute of Genetics, Biomedicum Helsinki, Helsinki, Finland; 6 Department of Genetics and Molecular Medicine, King’s College London, London, UK

## Abstract

Knowledge of the genomic variation among different strains of a pathogenic microbial species can help in selecting optimal candidates for diagnostic assays and vaccine development. Pooled sequencing (Pool-seq) is a cost effective approach for population level genetic studies that require large numbers of samples such as various strains of a microbe. To test the use of Pool-seq in identifying variation, we pooled DNA of 100 *Streptococcus pyogenes* strains of different *emm* types in two pools, each containing 50 strains. We used four variant calling tools (Freebayes, UnifiedGenotyper, SNVer, and SAMtools) and one *emm*1 strain, SF370, as a reference genome. In total 63719 SNPs and 164 INDELs were identified in the two pools concordantly by at least two of the tools. Majority of the variants (93.4%) from six individually sequenced strains used in the pools could be identified from the two pools and 72.3% and 97.4% of the variants in the pools could be mined from the analysis of the 44 complete *Str. pyogenes* genomes and 3407 sequence runs deposited in the European Nucleotide Archive respectively. We conclude that DNA sequencing of pooled samples of large numbers of bacterial strains is a robust, rapid and cost-efficient way to discover sequence variation.

Next generation sequencing (NGS) technologies have enabled progress in many biological research areas such as whole genome studies of various organisms^1^. Bacterial pathogens such as *Streptococcus pyogenes* (i.e. group A *Streptococcus*, GAS) are among the increasingly sequenced genomes; currently 45 complete GAS sequences have been recorded in the Genomes OnLine Database^2^. GAS is one of the most important human pathogens causing numerous non-invasive and invasive infections and post-infection sequelae including pharyngitis, impetigo, toxic shock syndrome, necrotizing fasciitis, rheumatic fever, and poststreptococcal glomerulonephritis. Comparative genomic studies are applied to various bacterial species to study the evolutionary history and genetic signatures that distinguish the strains^3^,^4^. Recently, 3615 genomes of M1 and 1125 genomes of M89 serotype GAS strains were sequenced to investigate the molecular factors that led to the resurgence of GAS epidemics in the past few decades^5^,^6^.

The cost of NGS technologies dropping significantly over the past decade has contributed to the growth in the availability of sequenced genomes. Nonetheless, population level studies of large sample sets are still cost prohibitive in many situations. Pooled sequencing (Pool-seq), in which only a single library containing a mixture of DNA from all the samples is sequenced, is one approach to reduce costs and speed up identification of sequence variation without significant loss of information^7^. A key question is whether Pool-seq can accurately detect sequence variation and correctly estimate the allele frequencies^8^,^9^.

Pool-seq has been successfully used in various areas of research such as Genome Wide Association Studies (GWAS)^10^, polymorphism discovery and allele frequency estimation^11^, population resequencing^12^, and genome evolution^13^. On the other hand, limitations of pooling strategies include loss of linkage disequilibrium information, difficulty of distinguishing between sequencing errors and low frequency alleles and bias in allele frequency estimation resulting from inaccuracies in pooled DNA concentrations^8^,^14^. The implications of these limitations will likely be inessential if Pool-seq is used to identify the genomic or protein sequences with minimal variability for choosing optimal candidates to be used in diagnostic assays or vaccine development, given large enough pool sizes and sequencing depth.

We assessed Pool-seq for identifying polymorphisms in 100 GAS strains relative to one of the publicly available complete genomes, the *emm* 1 type strain, SF370. The *emm*1 (serotype M1) type is one of the well-known GAS *emm* types that is mostly isolated from both human invasive infections and pharyngitis in high-income countries^15^. Variant analysis of 44 of the complete genomes and the publicly available sequence data from the European Nucleotide Archive (ENA) was also undertaken using this same reference strain. This study is the first to utilize Pool-seq to identify polymorphisms from different strains of GAS and the first to compare the efficacy of Pool-seq to microbial sequences in a large genomic archive such as ENA. Our results confirm the robustness and cost-efficiency of the Pool-seq approach for variant discovery especially when coupled with polyploidy aware variant calling tools.

## Results

### Pooled sequencing depth of coverage

**Figure 1:**
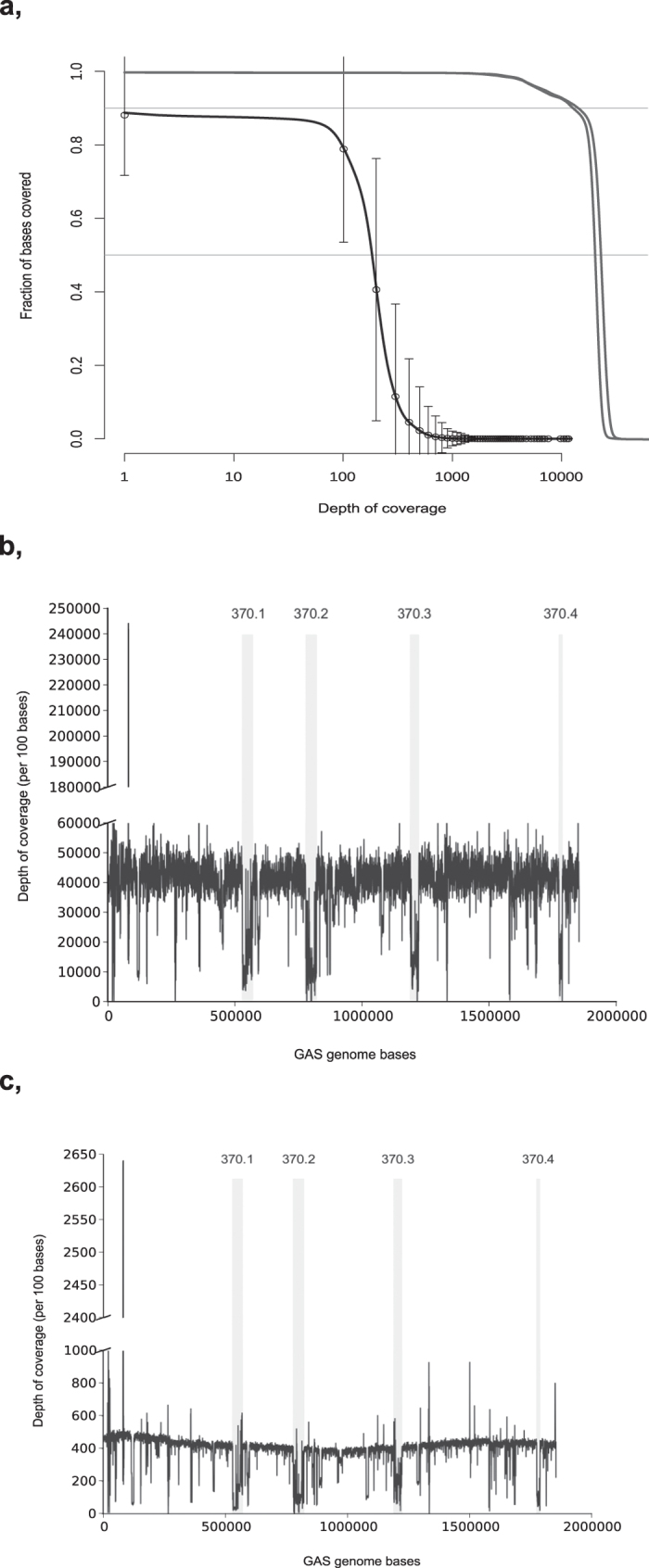
Sequencing depth of coverage for the pools and the public ENA runs. (**a**) The base coverage distribution from aligned data of the ENA runs and the two Pool-seq datasets. For the 3407 ENA runs the average is shown with ±1 s.d. In the two Pool-seq datasets ~90% of the bases had ~10,000X depth of coverage while in the ENA runs, the depth was on the average ~100X. (**b**) The aligned average depth of coverage of the two pools per 100 base length of the GAS genome. The positions of prophages of the SF370 strain are indicated by the gray fill and the prophage numbers (370.1 etc.). (**c**) The aligned average depth of coverage of the 3407 ENA runs per 100 base length of the GAS genome. The positions of prophages of the SF370 strain are indicated by the gray fill.

The aligned average depth for the two pools were ~18000x and 20000x. Compared to the individually sequenced publicly available ENA data, the two pools of the study had a much higher depth of coverage per strain (~400X vs ~186X) (Fig. 1a). The high depth could allow the use of a relatively higher lower limit (bound) for the minimum number of reads supporting the minor allele enabling separation of low frequency alleles from sequencing errors^8^. Therefore, we assessed the effect of various minimum bounds for allele calling using six individually sequenced strains (five strains from pool 1 and one strain from pool 2). We used a variant calling tool Freebayes^16^ to evaluate minimum bounds of 1%, 2%, 10%, and 50%. We were able to detect, on average, 97.0%, 96.5%, 83.7%, and 36.4% of the variants from the six strains at these thresholds, respectively. Given the high error rate of the sequencing platforms (ranging from 0.1 to 1% for Illumina^17^), we chose the 2% minimum bound for this study.

Next, we analyzed sequencing depth in each 100 bp stretch of the GAS genome. Certain regions of the genome were not covered by reads (approx. 0.23% of the GAS genome) while there were other areas with a high number of reads in both our pools and the ENA data (Fig. 1b,c). One of the regions with the highest depth of coverage (bases 81255 to 84157 of the SF370 reference genome) encodes a 23 S ribosomal RNA. Majority of regions with the lowest depth of coverage match with known prophage areas of the SF370 genome (Fig. 1b,c).

Various studies demonstrated the efficiency of Pool-seq for variant identification and allele frequency estimation at a much lower expected depth of coverage than that of the average depth in our study (for e.g. ref. 18 achieved > = 80% sensitivity at 40x). For this reason, we randomly selected 1% of the reads from one of our pools to investigate the impact of a lower depth of ~1000x (i.e. ~20x per strain) on the results. At this depth, on average, we were still able to mine ~94.9% of the variants from all the six individually sequenced strains. This indicates that we could have pooled clearly higher number of strains without significant loss of variant data.

### Identification and verification of genetic polymorphisms from the pools

To identify allelic variations in the two pools, we first mapped the sequence reads to the reference genome and then used four different variant calling tools, SAMtools^19^, Freebayes^16^, GATK’s UnifiedGenotyper^20^, and SNVer^21^. We decided to use more than one variant calling tool, owing to the reported low concordance between different variant calling methods^22^,^23^. We utilized variants identified from the six individually sequenced strains to evaluate the ability of each tool to call variants from the Pool-seq data. Prior to comparing the variants identified from the pools and the individual strains, multi-allelic and bi-allelic variant calls were decomposed and the variant positions were also normalized using the tool Vt^24^. Except SAMtools, all three tools identified more than 95% of the variants in all the individual strains (Table 1) and therefore proved to be suitable in calling variants from Pool-seq data.

Combining all the called variants from all the four tools slightly increased (by ~1%) the number of identified variants (Table 1) but since this approach is likely to increase the false positives, we chose to use more stringent options based on concordance of calls among the tools. Our final set of 63883 variants contain those that were concordantly identified by 2 or more of the tools relative to the reference strain (Table 2). Of these, ~28% fell in coding regions and had non-synonymous effect. Furthermore, three of the tools (Freebayes, UnifiedGenotyper and SNVer) could identify ~92.6% of the variants of the six strains concordantly.

Next, we studied the effect of duplicate removal on the variant calling since in whole genome re-sequencing studies, especially during variant calling, it is a common practice to remove both PCR and optical duplicates. Duplicate removal has also been recommended and applied in Pool-seq studies^7^,^24^. Existing software tools such as SAMtools and Picard regard reads that begin from the same position as duplicates. Inasmuch as the reads that have the same 5′ end position might not be PCR or optical duplicates, we called variants from our pools using SAMtools before and after duplicate removal to investigate the impact of duplicate removal on pooled samples. After duplicate removal, SAMtools failed to find 20% of the variants it identified before duplicate removal (Table 1).

**Figure 2:**
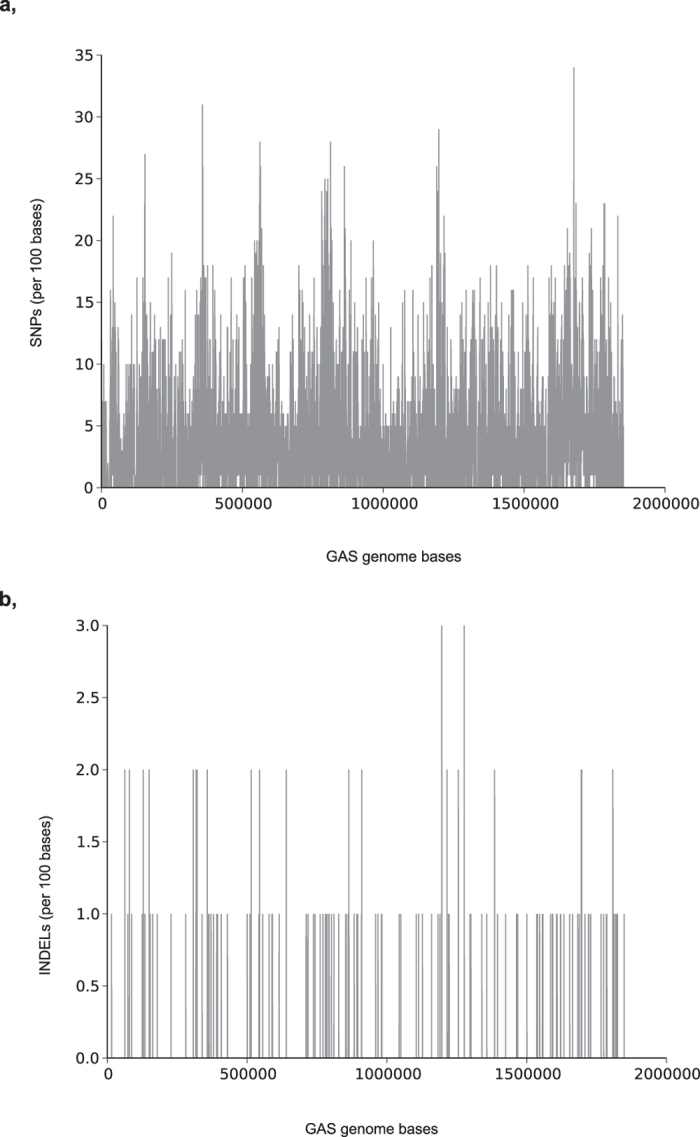
Distribution of the variants in the pools. (**a**) Distribution of the SNPs identified from the pools per 100 base length. (**b**) Distribution of the INDELs identified from the pools per 100 base length. The SNPs and INDELS are mostly uniformly distributed across the reference genome.

The SNPs and INDELs identified spread uniformly across the reference genome except in some areas that exhibited very high and low variations (Fig. 2a,b). Almost all the areas that had the largest number of SNPs (more than 27 per 100 bases) were in and around putative genes whose function has yet not been determined. To verify variants in some highly variable genes identified by the Pool-seq strategy, we selected two genes from the 20 most variable genes (*Spy_2009* and *Spy_0430*) for PCR and Sanger sequencing. Out of the 100 strains used for pooling, *Spy_2009* gene was amplified with PCR from 86% and the strains missing the gene were of *emm* types 6 and 12. However, only 30/86 strains gave an amplicon of the exact expected length (1250 bp) as judged by agarose gel electrophoresis. In the case of *Spy_0430*, PCR of only the *emm*1, *emm*78 and *emm*119 strains gave an amplicon. Therefore, the results indicate that some *emm* type strains in our pools lack these genes while some other strains exhibit high variability even in terms of the size of the gene.

**Figure 3:**
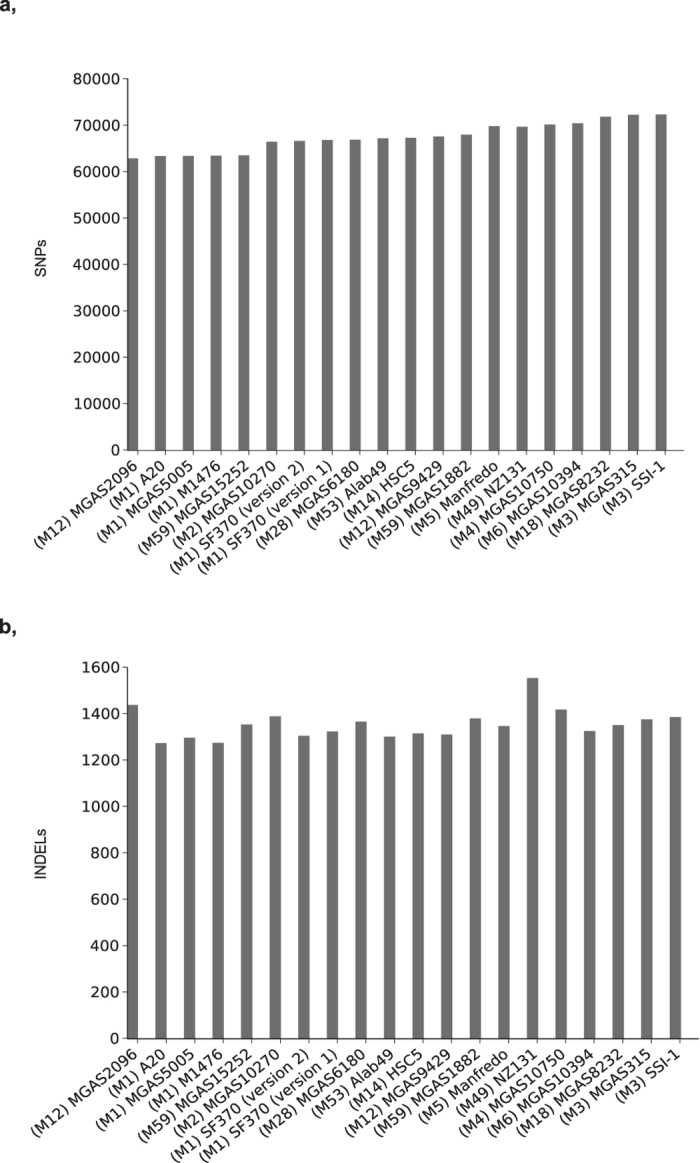
Variants from one of the pools compared to 20 of the publicly available complete GAS genomes. (**a**) Number of SNPs and (**b**) number of INDELs identified in one of the pools when 20 of the publicly available complete GAS genomes were used as a reference genome. Both the updated (version 2) and the previous version (version 1) of the SF370 genome are included.

To determine the effect in our results of choosing a particular reference genome, besides the SF370 updated genome (accession id AE004092), we also used 19 of the 45 complete genomes as a reference to analyze variants in one of the pools. The number of SNPs and INDELS from this analysis ranged from 62680 to 72133 and 1271 to 1551 respectively (Fig. 3a,b). This comparison showed that selection of the reference genome has a clear effect on the number of variants identified but that the effect is not drastic.

### Identification of genetic polymorphisms from public data

**Figure 4:**
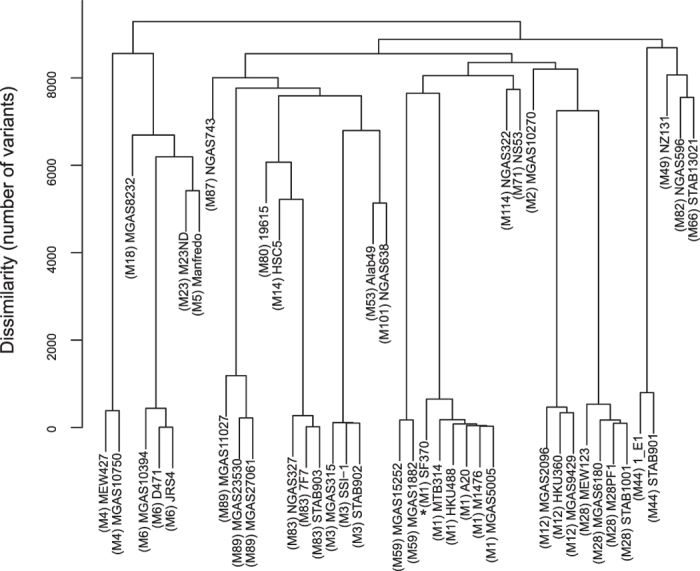
Hierarchical clustering of the 45 publicly available complete GAS genomes. The currently available complete GAS genomes are clustered based on the number of variants among them. The genome used as the reference genome in this study is marked with*.

The 44 complete genomes that were publicly available in September 7, 2016 were aligned to the selected reference genome. In total, 84312 SNPs and 3159 INDELs were discovered in all these genomes altogether (see Table 2). These variants were distributed similarly as those in the Pool-seq data. We further compared these 44 genomes and the reference genome with one another and clustered them based on the number of variants, which ranged from 6 to 9289 (Fig. 4).

To compare the variants identified from Pool-seq data to a larger dataset than the completed genomes available, we used GAS sequence data from the European Nucleotide Archive (ENA). We aligned to the reference genome initially 3513 paired end Illumina sequencing runs that had varying degrees of sequence quality (mapping percentage that ranged from 1% to 100%). We removed 106 of the runs that had mapping percentage of less than 1.5*IQR (<83.93% mapping percentage) from the variant analysis resulting in 3407 runs that had an average mapping and proper pair percentages of 93.3 and 91.0 respectively (Table 3). In this dataset there were 286502 variants containing 95.7% and 90.1% of the variants identified in the two pools and the 44 genomes, respectively (Table 4).

**Figure 5:**
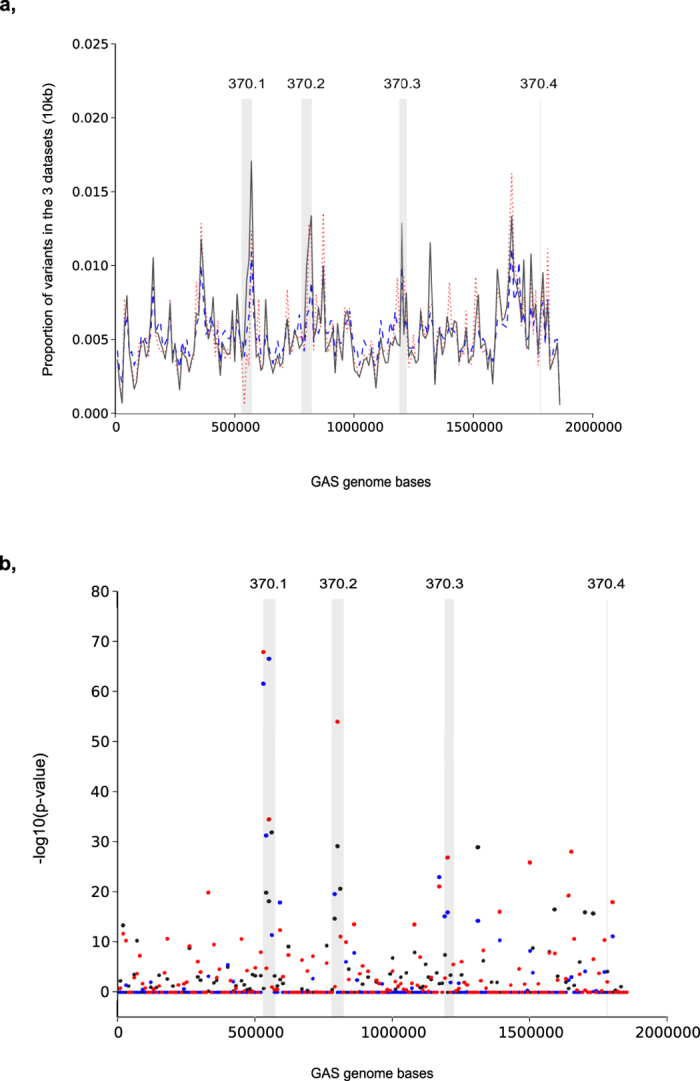
Relative variability of 10 kb regions in the 3 datasets. (**a**) Proportion of variants in 10 kb regions. The proportion has been calculated by a python script. Black solid lines represent the pools, red dotted lines the 44 genomes and blue dashed lines the ENA data. The positions of prophages of the SF370 strain are shown with gray fills. (**b**) Identification of the regions that show a statistically significant difference in the proportion of variants in the various datasets. The values are −log10 p-values from a fisher’s exact test with Bonferroni multiple correction. Black dots indicate the p-values of the proportions of variants between the pools and the ENA data, blue dots the p-values between the pools and the 44 genomes and red dots the p-values between the ENA data and the 44 genomes. The positions of the prophages of the SF370 strain are shown with gray fills.

To investigate whether certain regions were more variable in either the pools, the 44 genomes, or the ENA data, we divided the entire genome of the reference strain into 10 kb regions and calculated the proportion of variants in such regions (Fig. 5a). As analyzed using Fisher’s exact test, some areas appeared to have statistically significant different proportions of variants in these three datasets (Fig. 5b). There were two areas in particular where the 44 genomes showed the highest difference compared to the Pool-seq dataset and the ENA data. These were locations of the 370.1 and 370.2 prophages of the SF370 genome (Fig. 5b). Even though there were such areas that had differences in the proportion of variants, in general, based on the Welch test, the means of the proportions of the three sets were not statistically different (p-value 0.998).

## Discussion

In this report, we show that the Pool-seq strategy was successfully used in identifying variants in a large number of GAS strains. Furthermore, we show that data from large published datasets, such as the European Nucleotide Archive (ENA), can be used to identify variations in a wide range of GAS strains. Although all the data in this study pertains to GAS, it is likely that a similar approach could be utilized with other microbes too. Therefore, the results may have a significant impact on identification of variants in genes and proteins of various pathogenic microbes allowing rational selection of optimal, i.e. least variable, targets for diagnostic tests and vaccines. Additionally, the results can help in understanding genomic variation in GAS and lead to novel epidemiological tools based on next generation sequencing.

Understanding genomic variations that exist among bacterial strains might help delineate the distinctive nature of strains that, for instance, result in outbreaks of epidemic infections. Currently, such comparative studies are prevailing because of the growing availability of whole genome sequences of numerous bacterial strains. In GAS, various strains of both invasive and non-invasive nature have been whole genome sequenced. However, whole genome sequencing of individual strains may not be feasible due to time and money constraints and it is important to assess the robustness of Pool-seq as a potential alternative to sequencing strains individually. We have demonstrated that Pool-seq is a robust, rapid and cost-efficient alternative for genome-wide polymorphism surveys given high enough sequencing depth, suitable minimum read count, and the right choice of variant calling methods.

In Pool-seq, the precision of variant detection increases with the increase in depth of coverage and decreases with the minimum number of reads required for allele calling^8^. In this study, at the average aligned depth of ~20,000x, we were able to detect >90% of the variants found in the six individually sequenced strains with the false negative rate being at most ~10%. The false negative rate will be lower since the variants identified from the individual strains will not all be true variants. The variants that the Pool-seq approach failed to uncover had a very small average raw depth (in the tens range) compared to those that could be identified, which ranged in the tens of thousands. This indicates, similar to previous studies^25^,^26^, that the Pool-seq approach is not suited for studies that focus specifically on rare variants. Moreover, sampling bias during sequencing can be more pronounced in this approach due to unequal contributions of each sample to the resulting set of sequence reads.

The analysis with the random subset of reads showed that Pool-seq could be useful in variant discovery even at ~20x depth of coverage per strain than the average aligned depth that we reached (~400x per strain). In planning a Pool-seq approach, it is, however, necessary to be aware of an increase in the false negative rate by an increase in the required minimum proportion of reads. For example, the false negative rate can be up to ~63% at a minimum count threshold of 50%. We note that we used only a few individually sequenced strains for verifying the efficiency of Pool-seq and as a result the false negative rate could be higher for some strains due to differential representation in the pool. Further, because we had variant information for only six of the 50 strains in a pool, we cannot report the false positive rate from our study.

In our comparison of variant calling tools, using SAMtools with duplicate removal resulted in the identification of the least number of variants within each of the six individual strains. Some Pool-seq studies have used SAMtools and/or removed duplicates^11^,^27^. Mullen *et al*. used SAMtools for variant detection and removed PCR duplicates which resulted in identification of only 598 of the 1304 (45.9%) dbSNPs variants^11^. They attributed the high false negative rate to low coverage, lack of segregation of the SNPs and inaccurate dbSNP data. However, based on results from our study (on average 49.6% variants identified; see Table 1), we believe it is likely that the choice of SAMtools for variant calling and duplicate removal contributed to such a high false negative rate. We therefore recommend, at least for whole genome Pool-seq studies with high depth of coverage, to use variant calling tools that can handle pooled samples and to avoid duplicate removal. On the other hand, Gautier *et al*. observed for their Restriction site-Associated DNA (RAD) data that without duplicate removal the “effective pool size” substantially decreased, leading them to conclude that PCR duplicates considerably contribute to the overall experimental error^27^. Hence, in certain Pool-seq studies, such as those that employ RAD sequencing, duplicate removal might be appropriate. Further, methods that consider other base qualities besides read positions during duplicate removal, such as that employed by Chen *et al*.^28^, might be better suited in such circumstances.

Results from the analysis of the public data were used to confirm the efficiency of our pooling strategy by validating the variants we identified from the pools. For instance, we correctly identified low variability within the 23S rRNA region that is highly conserved among different strains of GAS. Similarly, we also found the prophage regions of the reference strain to have the lowest depth of coverage in our pools and the same was seen in the public ENA data. In our analysis of the public data, we also found that only a small portion of the variants identified from the ENA dataset was discovered in our two pools and the 44 complete genomes. This shows that the strains in our pools and the complete genomes represent only a small and geographically limited selection of strains. Therefore, if the Pool-seq strategy is used in identifying diagnostic or vaccine targets it is essential to select strains from as wide geographical area as possible.

In this study, we have examined issues such as the expected depth of coverage, representation of samples in a pool, frequency of alleles and variant calling methods that are central to a Pool-seq genome wide polymorphism detection study. We have demonstrated that Pool-seq can be an efficient and cost-effective alternative in polymorphism discovery for large samples of organisms, especially if a high quality reference genome is already publicly available.

## Materials and Methods

### Bacterial strains

The 100 GAS strains used in the two pools are listed in the supplementary material (Supplementary Table S1). The strains were selected from the bacterial culture collection of the National Institute of Health and Welfare so that each of the pools contained a similar wide array of *emm* types isolated in wide geographical area in Finland within years 1995–2012.

### DNA isolation, Pooling and sequencing

All strains were cultured overnight at +35 °C on blood agar plates in 5% CO_2_. DNA was isolated using UltraClean Microbial DNA Isolation Kit (MoBio) according to manufacturer’s instructions except for the following modifications: in the beginning 300 μl MicroBead solution and 6 μl mutanolysin (1 mg/ml) was mixed in a tube followed by addition of a 10 μl loopful of bacteria scraped from the culture plate. After incubation for 60 min at +37 °C, the solution was transferred to a MicroBead tube, and 2 μl of RNAse A (1 mg/ml) was added. From there, the manufacturer’s instructions were followed until at step 18, 35 μl of solution MD5 was added followed by 2 min incubation. Before pooling, the quality and the integrity of the DNA was checked using Nanodrop equipment (Thermo Scientific) and agarose gel electrophoresis. From each GAS strain 400 ng of DNA was used in one of the pools of 50 strains (Supplementary Table S1). The pools were precipitated and vaporized with SpeedVac and concentrations were measured with Qubit 62, 5 ng/μl and 86, 4 ng/μl for pool 1 and 2, respectively.

### Alignment and variant calling of the pools

Quality of the sequence reads from the pools were inspected using FastQC (version 0.11.2). Adapter and primer contaminants discovered by FastQC were trimmed using Trimmomatic (version 0.33)^29^. Bwa mem (version 0.7.10)^30^ was used for aligning the quality filtered reads to the reference genome using default parameters. The aligned read depth was obtained from the alignment bam files using Bedtools’ coveragebed utility (version 2.17.0)^31^. SAMtools (version 1.1), GATK’s UnifiedGenotyper (version 3.2–2), Freebayes (version 0.9.18-1) and SNVer (version 0.5.3) were used for variant calling. In SAMtools, the mpileup command was used with the maximum reads per input bam (–max-depth) of 10000 and a mapping quality of 20. INDEL realignment and base quality recalibration were applied before genotype calling by UnifiedGenotyper and a minimum phred-scaled confidence threshold of 20 for calling and emitting variants was used. For the BaseRecalibrator, the variants consensually identified by Freebayes and UnifiedGenotyper (variants after the first run before using BaseRecalibrator) were used. In Freebayes and SNVer, a variant was called if the minimum fraction of observations supporting the alternate allele was 0.02 and only bases of quality 13 or greater were counted. To generate an artificial lower depth (1000x) sample dataset from one of the Pool-seq datasets (pool 2), Seqtk was used for random selection of the reads.

### Sequencing of single strains

Six strains were cultivated overnight on sheep blood agar at 35 °C under 5% CO_2_, and DNA was extracted from bacterial colonies with MagAttract HMW DNA Kit (Qiagen). DNA extraction was done as the manufacturer indicates, but adding 6 μl mutanolysin (1 mg/ml) with lysozyme to improve DNA yield. Library was constructed with Illumina Nextera XT DNA Kit for sequencing and the library was sequenced with Illumina MiSeq 150 bp paired-end sequencing at Turku Centre of Biotechnology, Finland.

### Alignment and variant calling of the public data and the six individual strains

Reads from the ENA dataset and the six individual strains were quality checked using FastQC (version 0.11.2). Trimmomatic (version 0.33) was used to trim low quality reads and contaminants. Bwa mem (version 0.7.10), using default parameters, was used to align the quality filtered reads to the reference genome. Duplicates were marked using Picard (version 1.122). SAMtools (version 1.1) was used to call variants from reads having a mapping quality of at least 20.

The variants among the 45 complete genomes (including the used reference genome) were identified by using C-Sibelia (version 3.0.5)^32^ with default parameters.

### Analysis ensuing variant calling

The variants from the two pools were concatenated and merged using bcftools^19^. The variants from the 44 complete genomes and the ENA runs were merged in the same manner. Vt^24^ was used to normalize the variant calls before merging. SnpEff and SnpSift (version 4.0e)^33^ were used to annotate and filter the variants. Custom python scripts were employed for analyzing variant distributions throughout the reference genome and the protein coding regions.

### Statistical tests

Fisher’s exact test was used to compare the proportion of the variants from the pools, the ENA dataset and the 44 complete genomes. The p-values obtained were adjusted using the Bonferroni multiple test correction and a significance cut off value of 0.05 was used. The Welch Anova test was performed to compare the means of the proportions.

### PCR and Sanger sequencing verification of polymorphisms identified

Polymerase chain reaction and Sanger sequencing were used to confirm variant calling results. Two out of the 20 most variable genes were selected as the target genes and all samples from both the pools (50 samples in each pool) were investigated by PCR. DNA amplification was performed using GoTaq G2 Hot Start polymerase (Promega, USA) as the manufacturer indicates. The following primer pairs were used to amplify the investigated genes, respectively: 5′-TTTGATGAGGCAGCACATCT-3′ and 5′-TTTCAAAAGAAGGCATAGCAGT-3′; 5′-TAATGAGAGGAGTAAACTAGAAC-3′ and 5′-AGCCAAGAATTCACCAACCA-3′. Primers were designed to bind outside the gene region by using Primer3 software^34^. Heat cycling was done in T100 Thermal Cycler (Biorad) with the following program, initial denaturation at 95 °C for 2 minutes, 30 cycles at 95 °C for 30 seconds, 55 °C for 30 s, 72 °C for 70 s, and a final extension of 2 min at 72 °C. PCR product sizes were 1270 bp and 715 bp, respectively. PCR products were purified enzymatically by using Exo I and FastAP (Thermo Scientific) enzymes following manufacturer’s instructions. Purified PCR products were sequenced by Institute for Molecular Medicine (FIMM), Finland. Sequencing was done from both directions using the same primers as in PCR. Sequences were analysed using Geneious (version 7.1.9). Sequences from the different strains were aligned against each other and the reference genome.

## Additional Information

**How to cite this article:** Weldatsadik, R. G. *et al*. Sequence analysis of pooled bacterial samples enables identification of strain variation in group A streptococcus. *Sci. Rep.* **7**, 45771; doi: 10.1038/srep45771 (2017).

**Publisher's note:** Springer Nature remains neutral with regard to jurisdictional claims in published maps and institutional affiliations.

## Supplementary Material

Supplementary Information

## Figures and Tables

**Table 1:** Percentage of the variants identified from the six individual strains that were also mined from the pools using different variant calling tools and methods.

variant calling tools	Variants identified in individual strains (%)					
	strain1	strain2	strain3	strain4	strain5	strain6
SAMtools (with duplicate removal)	48.1	52.2	50.9	47.6	47.4	51.4
SAMtools (without duplicate removal)	67.2	71.0	71.2	69.9	63.4	70.0
Freebayes	96.9	97.0	96.9	96.8	95.7	96.5
GATK	95.0	94.9	94.9	94.9	92.2	94.3
SNVer	97.1	97.0	97.0	96.9	95.6	96.5
Union of the four tools	97.7	97.7	97.8	97.8	96.6	97.4
Freebayes + GATK + SNVer concordant	93.5	93.3	93.1	93.2	90.4	92.4
Concordant in 2 or more tools	94.1	94.1	93.9	93.9	91.2	93.4

Variants from strain1 were compared against pool 1 while the rest five strains were compared against pool 2. Where not mentioned, duplicates have not been removed.

**Table 2:** Total number of variants identified in the two pools, the 44 annotated genomes and 3407 runs from ENA.

	Two pools	44 GAS genomes	3407 runs from ENA
Total base count	40, 226, 329, 793	82, 600, 584	1, 529, 716, 686, 794
Total number of variants	63, 883	90, 321	286, 502
SNPs	63, 719	84, 312	270, 212
INDELs	164	3, 159	16, 290

**Table 3:** Alignment statistics of sequence reads from the two Pool-seq datasets and the ENA dataset formed from 3407 ENA runs.

	Two pools	3407 runs from ENA
Total number of reads	8.0*10^8^	1.4*10^10^
Mapped (%)	91.7	93.13 (83.9–100)
Unmapped (%)	8.2	6.8 (0–16)
Properly paired (%)	86.9	91.0 (78.0–99.6)

For the ENA dataset, besides averages, maximum and minimum values are given for proportion of the mapped, unmapped, and properly paired reads.

**Table 4:** Percentage of variants identified from the two pools, the 44 complete GAS genomes and the ENA dataset containing 3407 runs (rows) that were also found in the two other datasets(columns).

	Two pools	44 GAS genomes	ENA dataset
Two pools		72.3	97.4
44 GAS genomes	53.0		90.1
ENA dataset	21.9	27.6	

The reference strain SF370 was used in all three sets to identify the variants.
