# Supplementary material for: Sequence analysis of pooled bacterial samples enables identification of strain variation in group A streptococcus
Source: Sci Rep. 2017 Mar 31;7:45771. doi: 10.1038/srep45771 (PMC5374712; doi:10.1038/srep45771)
Supplement: Supplementary Information [file srep45771-s1.pdf]

Sequence analysis of pooled bacterial samples enables identification of strain variation in group A streptococcus

Rigbe G.Weldatsadik , Jingwen Wang , Kai Puhakainen, Hong Jiao, Jari Jalava, Kati Räisänen,

Neeta Datta, Tiina Skoog, Jaana Vuopio, T. Sakari Jokiranta, and Juha Kere

Supplementary Table S1. List of the GAS strains used in this study and their respective *emm* types

***emm* type**	**Pool 1**	**Pool 2**	**Total**
emm1.0	5	5	10
emm1.10	3	3	6
emm1.24	1	0	1
emm1.45	1	1	2
emm1.50	1	0	1
emm11.0	2	2	4
emm119.1	2	2	4
emm12.0	5	4	9
emm2.0	2	3	5
emm22.0	1	1	2
emm22.3	1	1	2
emm28.0	4	5	9
emm6.4	1	0	1
emm73.0	2	3	5
emm75.0	3	4	7
emm76.3	1	1	2
emm77.0	2	3	5
emm78.3	2	2	4
emm84.0	1	1	2
emm212 (st75.0)	1	1	2
emm11.1	1	0	1
emm4.0	1	1	2
emm78.0	2	2	4
emm89.0	5	5	10
**Total**	50	50	100
